# Diclofenac Concentrations in Post-Mortem Specimens—Distribution, Case Reports, and Validated Method (UHPLC-QqQ-MS/MS) for Its Determination

**DOI:** 10.3390/toxics10080421

**Published:** 2022-07-26

**Authors:** Paweł Szpot, Olga Wachełko, Marcin Zawadzki

**Affiliations:** 1 Department of Forensic Medicine, Wroclaw Medical University, 50345 Wroclaw, Poland; marcin.zawadzki@umed.wroc.pl; 2 Institute of Toxicology Research, 45 Kasztanowa Street, 55093 Borowa, Poland; olga.wachelko@iet.wroc.pl

**Keywords:** diclofenac, UHPLC-QqQ-MS/MS, abortion, distribution, fetus

## Abstract

The aim of the research was to establish a sensitive method for the quantification of diclofenac in postmortem samples. The developed method was applied in six cases: three fetuses in which the use of abortion pills by their mothers was suspected, one case of duodenal ulcer perforation, one case of traffic accident with fatal outcome, and one acute renal failure in which the distribution of diclofenac was examined. The analyses were performed using liquid–liquid extraction of postmortem samples and the quantification of diclofenac via ultra-high performance liquid chromatography, coupled with triple quadrupole tandem mass spectrometry. Gradient elution using a C18 column was applied. Electrospray ionization measurement in positive multiple reaction monitoring mode was used. Diclofenac-d4 was used as an internal standard. The validation parameters were as follows: lower limit of quantification: 0.5 ng/mL, linearity of calibration curve: 0.5–500 ng/mL, intra- and interday accuracies and precisions: not greater than 15%; recovery values: 72.0–102.2%, and matrix effect: 2.2–28.0%. The developed method enabled the determination of diclofenac in human postmortem biological fluids (blood, urine, vitreous humor, bile, and stomach content), tissues (placenta, kidney, liver, and heart), and in exhumated fetus bones, with high recovery, sensitivity, precision, and accuracy.

## 1. Introduction

Diclofenac is a nonsteroidal anti-inflammatory drug (NSAID) of the phenylacetic acid class used in human and veterinary medicine, due to its anti-pyretic, analgesic, and anti-inflammatory properties. Its major mechanism of action is the inhibition of cyclooxygenases COX-1 and COX-2, the basic enzymes in the biosynthesis of prostaglandins.

This drug is used by 7.6 million people yearly [1]. Diclofenac is considered to be safe; however, in terms of forensic toxicology, it may be important to its quantification in postmortem samples. Two main reasons are acute diclofenac intoxications [2] and anaphylactic shock after diclofenac ingestion. In Europe, severe anaphylaxis occurs in 1–3 per 10,000 people, with a mortality rate of 0.65–2%. In the United States and Australia, these rates are even higher [3]. Van Der Klauw et al. [4] identified 30 cases of anaphylaxis related to diclofenac in 773 reports on drug reactions. The studies conducted in Netherlands [5] showed that glafenine, amoxycillin, and diclofenac were the three most important causes of hospital admission after drug-induced anaphylaxis. The estimated incidence of diclofenac-induced anaphylaxis affects 1 per 10,000 to 20,000 patients. Examinations performed in Italy confirmed that diclofenac is the most frequently NSAID associated with anaphylaxis [6]. Similar conclusions have been also observed in other studies [7,8]. There have been a few reported single cases of anaphylactic shock after the administration of diclofenac: after oral ingestion [9,10,11,12], one after intravenous injection [13] and four after intramuscular injection [14,15,16], two cases after rectal administration [17,18], and one after received the diclofenac patch [18]. Picaud et al. [19] described nine cases of hypersensitivity to diclofenac registered by the Allergy Vigilance Network in France between 2002 and 2012. There was one case after intravenous administration, two cases after intramuscular injection, five cases after oral administration, and one after diclofenac contact with the ocular mucosa. The necessity of determining diclofenac in blood is also related to its ability for inducing: fatal hepatitis [20], Nicolau syndrome [21], fatal toxic epidermal necrolysis, acute immune hemolytic anemia [22], fatal necrotizing fasciitis [23], and rhabdomyolysis [24,25,26,27,28], which also can lead to death [27]. In some countries, pharmaceutical abortion is illegal. In order for pregnancy termination, Arthrotec^®^ (containing diclofenac and misoprostol) is often used. Therefore, detecting the presence of diclofenac in biological samples with other abortifacient substances (e.g., misoprostol) may be helpful for the investigation in cases where lawbreaking and crimes have been committed (e.g., intentional administration of Arthrotec^®^ to a pregnant woman by the child’s biological father) [28].

Determination of diclofenac may also be important for veterinary and environmental studies. This drug is used worldwide as a veterinary drug for domestic mammals, and therefore, it may pose a risk to many bird populations that are particularly vulnerable to its toxic effects. Diclofenac is particularly dangerous to raptors and vultures because of the presence of this drug in the livestock carcasses that these scavengers eat [29]. The mortality caused by diclofenac is the main cause of the observed population declines in some vulture species [30,31,32]. The estimated LD50 of diclofenac for some subgenre is only 0.1–0.2 mg/kg [29].

To quantify the diclofenac in biological samples, high-performance thin-layer chromatographic (HPTLC) [33,34], liquid chromatography with: a fluorometric detector [35], an electrochemical detector [36,37], a UV detector [38,39,40,41,42,43,44,45,46], gas chromatography–mass spectrometry (GC-MS) [47,48,49,50,51,52,53,54,55,56], electrospray ionization–ion mobility spectrometry [57], and liquid chromatography–mass spectrometry (LC-MS) [58,59,60,61] methods have been developed.

This paper aims to apply an ultra-high performance liquid chromatography–tandem mass spectrometry with triple quadrupole (UHPLC-QqQ-MS/MS) method for the determination of diclofenac in postmortem samples. The developed and fully validated method was applied for diclofenac quantification in biological fluids and tissues in six forensic cases, as well as in a postmortem distribution study. None of the presented cases were related to diclofenac intoxication. The authors decided to verify the method on authentic samples, and focused exclusively on diclofenac determination.

## 2. Materials and Methods

### 2.1. Chemicals and Reagents

Diclofenac, diclofenac-d4 (internal standard, IS), water (Chromasolv^®^ LC–MS), acetonitrile (Chromasolv^®^ LC–MS), methanol (Chromasolv^®^ LC–MS), ammonium carbonate, and formic acid were purchased from Sigma-Aldrich (Steinheim, Germany); ammonium formate was purchased from Sigma-Aldrich (Mumbai, India). Standard solutions of diclofenac and diclofenac-d4 were prepared in methanol. The standard solutions were stored in a refrigerator at −20 °C.

### 2.2. Instrumentation

Chromatographic analysis was performed using an ultra-high performance liquid chromatography system (UHPLC Shimadzu Nexera LC-40 System, Kyoto, Japan). The separation was performed using a Kinetex XB-C18 column (100 × 2.1 mm, i.d., particle size 2.6 µm; Phenomenex, Torrance, CA, USA) with a guard column, SecurityGuard Ultra C18 (15 × 2.1 mm; Phenomenex), with a thermostat at 40 °C. A mixture of 10 mM ammonium formate/0.1% formic acid in water (A) and 10 mM ammonium formate/0.1% formic acid in methanol (B) was used as a mobile phase. The gradient elution was carried out at constant flow, 0.3 mL/min. The gradient applied was as follows: 0 min. −5% B, 7.5 min −95% B, and then 10 min −95% B. A return to the initial gradient compositions (95% A and 5% B) was performed at 5 min. The injected volume was 2 μL.

Detection of the diclofenac was achieved using a triple-quadrupole mass spectrometer (QqQ, Shimadzu 8060, Kyoto, Japan). The spectrometer was equipped with an electrospray ion source (ESI); determination of the diclofenac was carried out in multiple reaction monitoring (MRM) mode. The following MS parameters were fixed: nebulizing gas flow: 3 L/min, heating gas flow: 10 L/min, interface temperature: 300 °C, DL temperature: 250 °C, heat block temperature: 400 °C, and drying gas flow: 10 L/min. A summary of the precursor and product ions, collision energies, dwell time, Q1–Q3 pre bias voltages, and retention time for each compound is presented in Table 1. The most optimal collision energies (CE) were selected using the MRM method optimization software. The same procedure was applied to diclofenac-d4. Under the chromatographic conditions, the *m*/*z* transitions of 297.3→ 214.3, 297.3→ 216.3, 297.3→ 252.1, and 301.5 → 220.2, 301.5 → 218.3 were selected for the optimal monitoring of diclofenac and diclofenac-d4, respectively.

### 2.3. Blank Samples

Blank samples of postmortem human blood, bile, placenta, urine, kidney, liver, and stomach content were collected during autopsies performed in the Department of Forensic Medicine. Blank samples were screened prior to spiking, to ensure that they were free from diclofenac. Authentic biological samples collected in six forensic cases were sent to our laboratory for routine toxicological analyses of psychoactive substances and pharmaceuticals. Biological fluids were collected in tubes with sodium fluoride, and solid tissues were collected in plastic containers (without any preservative agent).

### 2.4. Case Reports

Case 1: A female fetus (338 g, 27 cm, 20–21 weeks pregnancy) was found at a sewage farm. The fetus was connected by an umbilical cord to the placental tissue. There were no features of live birth. The umbilical cord was wrapping the fetus body, especially its neck, which may indicate that the cause of intrauterine death was asphyxiation.

Case 2: A man was found dead. The cause of death of the deceased was acute renal failure. The detected diclofenac did not affect death.

Case 3: An unidentified male was found dead in bed at home. The cause of death of the deceased was a perforation of a duodenal ulcer, with diffuse peritonitis.

**Figure 1:**
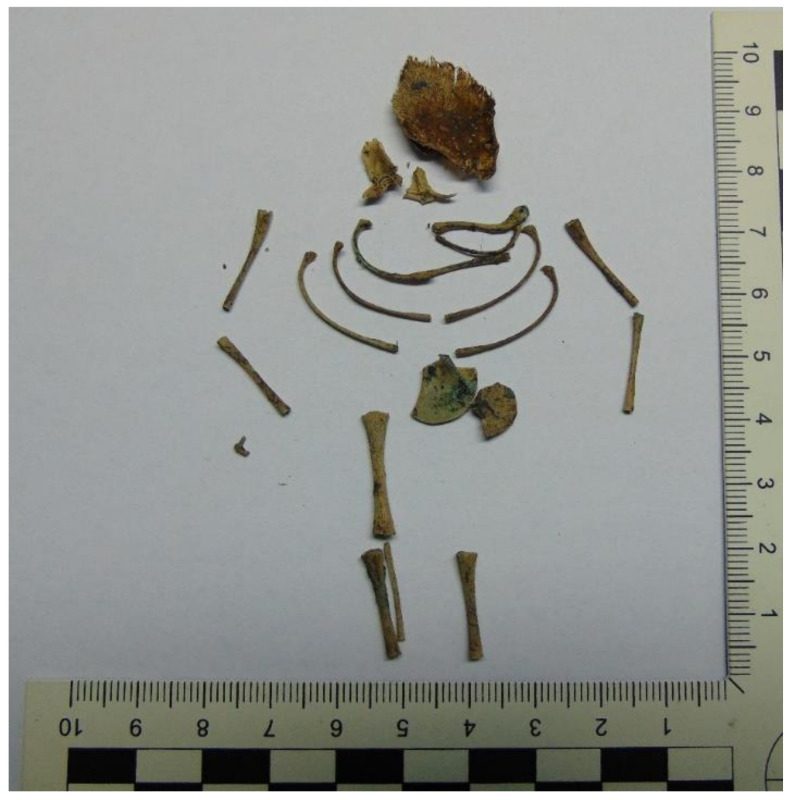
The remains of a human fetus (case 4).

Case 4: A female fetus, age approximately 16 weeks, was exhumed approximately 8 months after burial (Figure 1). After a miscarriage, the mother decided to surround the fetus with a towel and leggings. Further, she put the body and the blood-stained sanitary pad into a plastic bag and buried it about 0.5 m under the ground next to the house. The cause of fetus death was unknown. Fetal bones and a sanitary pad were provided for toxicological examinations.

Case 5: A female fetal corpse (290 g, 29 cm, 18–22 weeks pregnancy) in a state of advanced putrefaction was found on a balcony in a plastic pot. The corpse was covered with a brown colored blanket and placed in a plastic bag. The suspect mother of the child stated that she had miscarried approximately 4–5 months earlier.

Case 6: A woman died as a result of a traffic accident. The cause of death was multi-organ injuries.

For further toxicological analysis, biological fluids and tissues were collected. In case 1: blood and placenta; in case 2: blood, urine, vitreous humor, stomach content, bile, kidney, and liver; in case 3: blood, urine, and vitreous humor; in case 4: bones and bloodstained sanitary pad (only for qualitative analysis); in case 5: liver and heart; in case 6: blood and vitreous humor. Each of the liquid samples (blood, urine, vitreous humor, and bile) were collected in a tube containing sodium fluoride as the preservative agent.

### 2.5. Working Solutions, Calibration Curve, and Quality Control Samples

Standard solutions were diluted with methanol to obtain working standard solutions at the following concentrations of diclofenac: 5, 10, 50, 100, 500, 1000, 2000, and 5000 ng/mL. Calibration points and quality control samples (QC) were prepared by mixing the diclofenac working solutions with the human postmortem samples. The final diclofenac concentrations were as follows: 0.5, 1, 5, 10, 50, 100, 200, and 500 ng/mL (biological fluids) or ng/g (solid tissues). Quality control samples were prepared by spiking blank human postmortem samples to yield final concentrations of 1 (low QC), 50 (medium QC), and 500 (high QC) ng/mL or ng/g for diclofenac.

### 2.6. Sample Preparation

Human postmortem blood (200 μL) was transferred into 12 mL plastic vials. Next, 20 μL of methanolic internal standard solution (diclofenac-d4 at a concentration of 100 ng/mL) was added, along with 200 μL of buffer (0.5 M ammonium carbonate, pH 9). Liquid–liquid extraction (LLE) with 2 mL of ethyl acetate was carried out for 10 min. The samples were centrifuged for 10 min (at 2540× *g* at 4 °C). The organic phase was transferred into 2 mL Eppendorf tubes and evaporated to dryness under a stream of inert nitrogen gas (at 40 °C). The dry residues were dissolved in 50 μL of methanol. The solution was then transferred into glass inserts for autosampler vials and analyzed via ultra-high performance liquid chromatography–triple-quadrupole tandem mass spectrometry (UHPLC–QqQ-MS/MS).

The biological materials such as urine, vitreous humor, bile, and stomach content were prepared as human postmortem blood. Because the concentrations of diclofenac in some cases were markedly above ULOQ (500 ng/mL), the assay was repeated. Samples were diluted with water (LC-MS grade) 100-fold.

A total of 1 g of solid tissue (liver, kidney, placenta) was transferred to 12-mL plastic tube. Next, 1 mL of water (Chromasolv^®^ LC–MS) was added, and the sample was homogenized using an Q55 sonicator (QSonica, Newtown, CT, USA). Next, 200 µL of the homogenate was subjected to the same procedure as postmortem blood.

Dry bones (case 4), prior to extraction, were homogenized using a ball mill LMK-s (Testchem, Radlin, Poland). To the 50 mg bone homogenate, 200 µL of LC-MS grade water was added, and the mixture was placed in an ultrasonic bath for 60 min. Next, the sample was allowed to stand in the fridge (4 °C) for 24 h. The homogenate was subjected to the same procedure as the postmortem blood.

Bloodstains on the sanitary pad were cut out in several places (0.5 × 0.5 cm) with scissors, flooded with water, and treated in an ultrasonic bath for 60 min. Next, the sample was allowed to stand in the fridge (4 °C) for 24 h. The supernatant was prepared using the same procedure as the postmortem blood. A qualitative analysis was performed.

### 2.7. Method Validation

The evaluated parameters of the method included an examination of selectivity, linearity, precision, and accuracy, the lower limit of quantification and recovery, and the matrix effect. The validation of the method was performed in accordance with GTFCh (Gesellschaft für Toxikologische und Forensische Chemie ang. *German Society of Toxicological and Forensic Chemistry*) recommendations.

#### 2.7.1. Selectivity

Blank blood, bile, placenta, urine, kidney, liver, and stomach content were tested for possible endogenous interference peaks at the retention time of the diclofenac.

#### 2.7.2. Linearity

Linearity was evaluated using an analysis of the diclofenac working solution with human postmortem biological matrix at final concentrations of 0.5, 1, 5, 10, 50, 100, 200, and 500 ng/mL or ng/g. The coefficient of determination (R2) was determined. According to the acceptance criteria used, the coefficient of determination should meet the condition: R2 ≥ 0.995. A linear calibration model was applied. 

#### 2.7.3. Precision and Accuracy

The precisions and accuracies of the method were estimated by replicating the analysis (*n* = 5) of QC samples at three concentration levels: 1 (low QC), 50 (medium QC), and 500 (high QC) ng/mL or ng/g. The precision and accuracy were expressed as RSD% (relative standard deviation) and RE% (relative error), respectively.

#### 2.7.4. Lower Limits of Quantification (LLOQ)

The lower limit of quantification (LLOQ) was defined as the minimal concentration at which the RSD% does not exceed 20%.

#### 2.7.5. Recovery and Matrix Effect

The recovery (*n* = 5) of the diclofenac was evaluated at each of the three concentrations of QC (1, 50, and 500 ng/mL or ng/g). The recovery (%, *n* = 5) was determined by comparing the response of extracted analyte in spiked blank matrix with the response of the analyte spiked after the extraction of the blank matrix. The matrix effect (%bias, *n* = 5) was determined by comparing the response of the analyte spiked after the extraction of blank matrix with the response of the analyte in neat solution. Matrix effects and recovery values were calculated using equations described by Chambers et al. [62].

## 3. Results

### 3.1. Method Development

**Figure 2:**
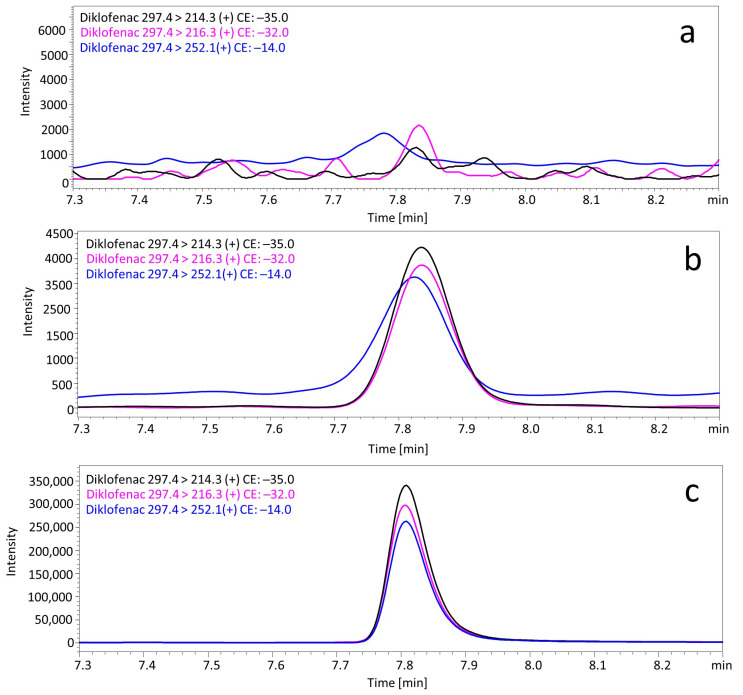
MRM (multiple reaction monitoring) chromatograms of diclofenac in (**a**) blank human postmortem blood, (**b**) low QC: 1 ng/mL, and (**c**) real sample of human blood (case 3).

A simple liquid–liquid extraction was successfully applied to extract the diclofenac and the IS from postmortem samples. No interfering ion current signals were observed at the retention time of diclofenac (Figure 2a). The linear concentration range was from 0.5 to 500 ng/mL or ng/g. The coefficient of determination (R2) was > 0.997 for all matrixes. The analysis of a sample containing 1000 ng/mL or ng/g of diclofenac resulted in the saturation of the detector. A LLOQ of diclofenac in human postmortem samples was determined to be 0.5 ng/mL. The recovery and matrix effects, and the intra- and interday precision and accuracy values for all postmortem matrixes are presented in Table 2. The intraday RSD% data obtained from five repetitive measurements of samples at three concentration levels (1, 50, and 500 ng/mL or ng/g of diclofenac) ranged from 0.8% to 13.1%. The interday RSD% ranged from 0.3% to 14.6%. The intra- and interday accuracies at the three quality control levels did not exceed the value of 15.0%. Based on the above results, it can be concluded that the method is sufficiently accurate and precise to be used in routine forensic toxicological analysis. The mean recovery values were in a range from 72.0% to 102.2%. Regarding the matrix effects, all concentrations ranged from 2.2% to 28.0% of the nominal values, suggesting that there were no significant matrix effects in diclofenac determination. 

### 3.2. Diclofenac Concentrations in Biological Samples

In case 1, diclofenac was measured in blood at concentration of 429.4 ng/mL and in placenta at a concentration of 1036.7 ng/g. Diclofenac concentrations in biological materials from case 2 were as follows: blood (108.2 ng/mL), vitreous humor (10.7 ng/mL), bile (14931.1 ng/mL), urine (82.4 ng/mL), stomach content (229.1 ng/mL), liver (50.5 ng/g), and kidney (153.8 ng/g). In case 3, the diclofenac concentrations were: blood (121.7 ng/mL), vitreous humor (37.8 ng/mL), and urine (12631.3 ng/mL). In case 4, diclofenac was detected at concentration of 50.0 ng/g in exhumated fetus bones. The qualitative analysis of bloodstains from a sanitary pad also found the presence of diclofenac. Diclofenac concentrations in liver and heart in case 5 were 6938.0 ng/g and 6585.0 ng/g, respectively. In case 6, the analysis revealed diclofenac at a concentration of 207.2 ng/mL in blood and 15.1 ng/mL in vitreous humor. The determination of diclofenac in vitreous humor and heart was performed on urine and liver calibration curves, respectively. The quantification of diclofenac in exhumated fetus bones was performed using the isotope dilution method. The summarized analysis results are presented in Table 3.

## 4. Discussion

Methods using HPLC without a mass spectrometry detector show low sensitivity and selectivity (the presence of interfering peaks could lead to the interpretation of false results). In turn, GC-MS methods often require large amounts of biological material and the derivatization process, which is complex and time-consuming. The exception is one previously published article, in which 200 µL of blood was used, and no derivatization was needed [56]. The comparison of LC-MS methods for the determination of diclofenac in biological samples is shown in Table 4. In the summarized studies, a liquid chromatography coupled with a triple quadrupole spectrometer or an ion trap spectrometer was used. One article described the determination of diclofenac in bovine milk [58], one in microdialysis samples [63], one in rat skin [64], one in dairy cow plasma [59], one in rabbit plasma [60], one in mouse plasma [61], one in rat plasma [65], one in fish plasma [46], and three in human plasma [66,67]. The fact that most of the methods in Table 4 used plasma as the matrix is most understandable, as the methods were developed to control diclofenac concentrations in clinical toxicology practice and in pharmacokinetic studies. In four articles [58,61,64,66] an isotope-labeled standard of diclofenac has been used as the internal standard. The most popular method of sample preparation was protein precipitation [59,60,61,65,68,69] followed by liquid–liquid extraction (LLE) [58,64,66,70]. The most sensitive method (LOQ: 0.05 ng/mL) was developed by Nazario and Lancas [58]; however, the authors used two steps for LLE, which is complicated and time-consuming. Furthermore, they tested bovine milk, which is not as complex a biological matrix as postmortem biological fluids and tissues. In addition, the sample volume was 2000 µL; therefore, it could be hard to apply this technique for forensic purposes. The injection volume was also 15-fold greater than in our method.

It is difficult to compare methods using different matrices and different sample volumes. In forensic toxicology, plasma or serum is rarely available for testing (due to the putrefaction of biological material and the hemolysis of blood), so it is especially important to have a precise, sensitive, and accurate method for the determination of diclofenac, strictly in the case of the toxicological analysis of postmortem biological fluids and tissues. To date, there have been only four LC-MS methods introduced that involve human blood as a matrix of diclofenac quantification [68,69,70,71]. However, three of them are multi-compound methods that do not focus on the determination of diclofenac alone in the blood. The limits of quantification are quite high: 60 ng/mL [69], 100 ng/mL [70], and 500 ng/mL [68], and therefore, these methods are not suitable for trace analysis. In turn, the multi-component method described by Al-Asmari [71] is based on a solid-phase extraction (SPE) procedure that requires up to 1000 µL of the sample. In addition, in most of the applied methods, the isotope-labeled diclofenac was not used as an internal standard. This may be the main reason for the poor recovery values in the method described by Di Rago et al. [70]. The development of a sensitive method for the determination of diclofenac in postmortem blood is important, especially in cases when death as a result of sepsis, multi-organ failure, fatal hepatitis, or rhabdomyolysis is expected, and the autopsy is performed several days (6–24 days) after intoxication [21,27]. A low amount of postmortem blood is especially important in examinations of samples collected from newborns or miscarried fetuses, because of the difficulties in obtaining large amounts of biological specimens. It should be added that the diclofenac concentration in postmortem material has been determined in none of the literature-reported fatal cases related to complications after the use of diclofenac. In particular, the examination of the postmortem blood of fetuses is very rare. For this reason, the three cases from toxicology practice presented in this paper may provide valuable new information.

The biological samples collected in case 1, case 4, and case 5 were also tested for abortifacients; however, an analysis did not reveal the presence of such substances. Cases 1 and 4 are interesting, due to the fact that they concern fetuses examinations. The first shows that the concentration of diclofenac is higher in the placenta than in the fetus blood sample, which suggests that the placenta may be a better material for exposure studies in the case of diclofenac determination. In addition, the results obtained in case 4 prove that there is a possibility for the quantitative analysis of diclofenac, even in exhumed samples such as bones. The use of abortion pills is illegal in some countries; therefore, the termination of pregnancy may only be performed in hospitals by medicinal staff. However, in some cases, pregnant women purchase abortion pills from the Internet. Mifepristone and misoprostol are the substances most frequently used for the effective termination of pregnancy. The easiest obtainable source of misoprostol are drugs for arthritis: Arthrotec^®^ and Arthrotec forte^®^. One tablet of Artrotec^®^ contains 50 mg of diclofenac with 0.2 mg of misoprostol, while Arthrotec forte^®^ contains 75 mg of diclofenac with 0.2 mg of misoprostol. Misoprostol and its metabolite misoprostol acid cause a uterine contraction, resulting in premature labor [28]. Due to the very low doses, the rapid metabolism of misoprostol, and the high instability of misoprostol acid, it is very difficult to detect these substances in the biological material collected from fetuses. In such cases, diclofenac is usually found alone, especially with the fact that diclofenac is present in Arthrotec^®^ at a much higher dose. It is also more stable in biological material in comparison to previous described compounds. The detection of diclofenac in the samples collected from the fetuses is not indicative of a pharmacological abortion with the use of Arthrotec^®^. The detection of this substance only confirms that the mothers were taking drugs containing diclofenac before the abortion or the miscarriage.

In the case 2, there were many different types of biological material collected during the autopsy; therefore, the distribution study was performed. The presented research findings shows that the lowest concentration of diclofenac can be observed in the vitreous humor, while the highest is in the bile. The literature indicates that 60% of diclofenac is excreted into the urine. The relatively low concentration of diclofenac in urine in case 2 can be explained by the specificity of the incident circumstances. In the described case, acute renal failure was found. Renal malfunction probably affected the urinary excretion of diclofenac.

The man’s death in case 3 was related to the perforation of the duodenal ulcer with diffuse peritonitis. It is not possible to establish whether the duodenal ulcer was induced via the ingestion of diclofenac, or whether the deceased had already struggled with the disease previously. However, it is worth noting that diclofenac should not be used in the above-mentioned conditions. Medical history and witness statements were not available in this case, so it cannot be excluded that chronic NSAID ingestion was the cause of duodenal ulcer formation. In contrast to case 2, diclofenac in the urine in this case is very high, exceeding 12 µg/mL. In cases 2 and 6, it is noticeable that the concentration of diclofenac in the vitreous is only about a few percent of the blood concentration. Even in case 3, where the urinary concentration of diclofenac is above 10,000 ng/mL, its concentration in the vitreous is relatively low.

Kerr and Fletcher reported a case of suicide by poisoning with nefopam [72]. A 19-year-old male was brought into the emergency department after the ingestion of 60 tablets (30 mg) with nefopam hydrochloride and 14 tablets (50 mg) of diclofenac sodium. Postmortem toxicology examinations revealed a serum nefopam concentration of 7.45 µg/mL and an extremely high diclofenac concentration of 69 mg/mL. The confirmed cause of the man’s death was nefopam overdose. McIntyre et al. [73] described an interesting case of multi-drug poisoning. The mentioned report revealed the presence of least nine discernible pills (diclofenac) in the stomach and duodenum. Diclofenac was not detected in the peripheral blood or liver, but the gastric content contained approximately 1100 mg of this substance. These data suggest that diclofenac was ingested shortly before the death and was not absorbed. However, in this case, more than one hour elapsed between the ambulance call and the death confirmation; therefore, it is more likely that the method for diclofenac determination used by the authors was not sensitive enough. Fels et al. [74] reported two cases of U-47700 poisoning in which diclofenac in the blood was also presented at the following concentrations: 15 ng/mL (heart blood, 35-year-old male) and 25 ng/mL (femoral blood, 45-year-old male). The blood concentration of diclofenac in the cases described in this paper (108.2–429.4 ng/mL) are considerably lower than in the suicide attempt reported by Kerr and Fletcher. However, this may be explained by the fact that neither of the reported cases were related to the intentional taking of one’s own life with the use of diclofenac.

The method described in this presented paper can also be applied in veterinary toxicology. Shultz et al. [32] determined diclofenac in the kidneys and livers of birds to be at concentrations ranging from 4 to 160 ng/g. Such concentrations can be successfully determined with the use of the presented UHPLC-QqQ-MS/MS technique.

## 5. Conclusions

A sensitive method based on liquid–liquid extraction (LLE) of the sample and UHPLC–QqQ-MS/MS analysis was developed to quantify diclofenac in postmortem blood samples, with a low limit of quantification as well as very good recovery, precision, and accuracy values. The developed method was successfully applied for the examination of postmortem samples in six cases. The distribution of diclofenac in seven biological materials was investigated. It has been proven that the presented method can be successfully used in the routine forensic examination of diclofenac concentrations, even in putrefied blood samples and exhumated specimens. The importance of our study for the future research is based on the fact that the application of simple LLE (pH 9) is an optimal solution for the determination of diclofenac in a wide range of biological samples, even in the case of very complex matrices (solid tissues) and completely non-routine analyses of trace diclofenac residues in exhumed bones. Reports of postmortem blood levels of diclofenac are rare in the literature. The possible reason for this is that diclofenac ingestion usually does not lead to the fatal intoxication. However, it should be considered that the adverse effects that diclofenac can cause (anaphylactic shock, hepatitis, toxic epidermal necrolysis, acute immune hemolytic anemia, necrotizing fasciitis, and rhabdomyolysis) are potentially fatal. In order to establish a direct relationship between diclofenac ingestion and fatal anaphylactic shock or rhabdomyolysis, the presence of diclofenac in the biological material of the deceased should be proven. Incorrect drug dosing by medical personnel, or inaccurate information in the medical history has been noted in previously reported cases. The method presented in this study can confirm that the described incidents occurred as a result of diclofenac ingestion.

## Figures and Tables

**Table 1:** UHPLC–ESI-QqQ-MS/MS parameters for diclofenac and diclofenac-*d_4_*.

Compounds	Retention Time (min.)	Precursor Ions (*m/z*)	Product Ions (*m/z*)	Dwell Time (msec)	Q1 Pre Bias (V)	CE (V)	Q3 Pre Bias (V)
Diclofenac	7.807	297.3	214.3 216.3 * 252.1	17	−11 −15 −15	−35 −32 −14	−22 −23 −16
Diclofenac-*d_4_*	7.794	301.5	220.2 218.3 *	17	−17 −18	−37 −34	−14 −14

* Ions selected for quantitative analysis.

**Table 2:** Recoveries, matrix effects, intra- and interday precision, and accuracy of diclofenac from postmortem samples using UHPLC-QqQ-MS/MS.

Biological Matrix	Validation Parameters									
	The Linear Concentration Range [ng/mL] or [ng/g]	The Coefficient of Determination (R^2^)	LLOQ [ng/mL] or [ng/g]	Concentration Level [ng/mL] or [ng/g]	Intraday		Interday		Recovery [%] *	Matrix Effect [%] *
					Precision [%] *	Accuracy [%] *	Precision [%] *	Accuracy [%] *		
Bile	0.5–500	>0.998	0.5	1 50 500	2.0 3.5 1.2	−4.3 13.8 5.7	10.1 0.9 1.8	−0.2 7.7 9.1	73.5 94.1 90.7	−26.5 −5.9 −9.3
Blood	0.5–500	>0.998	0.5	1 50 500	10.7 1.6 2.7	−12.7 7.9 −4.0	1.0 2.8 0.3	−5.0 7.7 −0.7	102.2 92.6 93.7	2.2 −7.4 −6.3
Placenta	0.5–500	>0.997	0.5	1 50 500	2.8 7.6 8.7	8.3 14.0 11.2	11.6 7.9 11.1	3.3 11.6 9.4	79.5 95.9 93.1	−20.5 −4.2 −6.9
Urine	0.5–500	>0.997	0.5	1 50 500	0.8 10.8 8.1	−9.5 2.2 −3.4	2.4 9.8 0.8	1.0 −3.9 −6.9	72.0 84.2 80.1	−28.0 −15.8 −19.9
Kidney	0.5–500	>0.999	0.5	1 50 500	2.2 4.2 11.9	10.7 2.7 2.9	14.6 2.4 5.0	14.9 −2.3 1.0	84.8 85.1 86.1	−15.2 −14.9 −13.9
Liver	0.5–500	>0.999	0.5	1 50 500	1.2 3.6 4.3	−3.8 2.8 12.0	2.2 4.5 5.5	2.0 8.3 2.0	74.5 89.7 90.3	−25.5 −10.3 −9.7
Stomach content	0.5–500	>0.997	0.5	1 50 500	13.1 3.9 5.0	12.0 2.3 1.2	0.7 0.5 6.0	14.5 −9.4 2.1	85.2 82.0 85.8	−14.8 −18.0 −14.2

* *n* = 5.

**Table 3:** Diclofenac concentrations in authentic forensic cases (biological fluids and tissues).

		Concentrations of Diclofenac [ng/mL ^a^ or ng/g ^b^]					
		Case 1 (Female Fetus)	Case 2 (Male)	Case 3 (Male)	Case 4 (Female Fetus)	Case 5 (Female Fetus)	Case 6 (Female)
Biological Fluids ^a^	Blood	429.5	108.2	121.7	─	─	207.2
	Vitreous humor	─	10.7	37.8	─	─	15.1
	Urine	─	82.4	12 631.3	─	─	─
	Bile	─	14 931.1	─	─	─	─
	Stomach content	─	229.1	─	─	─	─
Solid Tissues ^b^	Liver	─	50.5	─	─	6938.0	─
	Kidney	─	153.8	─	─	─	─
	Heart	─	─	─	─	6585.0	─
	Bones	─	─	─	50.0	─	─
	Placenta	1036.7			─	─	

─ material was not collected; ^a^ concentration for biological fluids; ^b^ concentration for solid tissues

**Table 4:** Comparison of LC-MS methods for determination of diclofenac in biological samples.

Biological Sample (Volume)	Sample Preparation	Instruments (Mode)	Recovery [%] /Internal Standard	LOQ [ng/mL] (Injection Volume)	References
Fish plasma (500 µL)	SPE	ESI-HPLC-QqQ-MS/MS (SRM)	76.0 ^13^CD_3_-labeled naproxen	─ (10 µL)	[46]
Bovine milk (2000 µL)	Two steps LLE (ethyl acetate)	ESI-UHPLC-QqQ-MS/MS (MRM)	85.5−89.1 diclofenac-*d_4_*	0.05 (30 μL)	[58]
Dairy cow plasma (200 μL)	Protein precipitation with ACN HCOOH	ESI-HPLC-QqQ-MS/MS (MRM)	97.6−101.8 tolfenamic acid	5 (5μL)	[59]
Rabbit plasma (100 µL)	Protein precipitation with ACN	ESI-UHPLC-QqQ-MS/MS (MRM)	54.1−67.1 flufenamic acid	80 (10 µL)	[60]
Mouse plasma (10 µL)	Protein precipitation with ACN	ESI-HPLC-QqQ-MS/MS (SRM)	89.0−103.0 diclofenac-*d_4_*	20 (30 µL)	[61]
Ringer-microdialysis samples (25 µL)	Dissolution in methanol and formic acid	HPLC/-QqQ-MS/MS (SRM)	─ indomethacine	1 (20 µL)	[63]
Rat skin (50 μL of enzymatically treated and homogenized sample)	LLE (methyl *tert*-butyl ether)	ESI-HPLC-QqQ-MS/MS (MRM)	64.5−68.4 diclofenac-*d_4_*	200 ^b^ (5 µL)	[64]
Rat plasma (50 µL)	Protein precipitation with MeOH	ESI-HPLC-QqQ-MS/MS (MRM)	─ naproxen	─ (7 µL)	[65]
Human plasma (1000 µL)	LLE (cyclohexane: *tert.* butylmethyl ether)	ESI-HPLC-QqQ-MS/MS (MRM)	─ diclofenac-*d_6_*	0.15 (50 µL)	[66]
Human plasma (500 µL)	SPE	ESI-HPLC-MS (SIM)	84.9 ─	100 ^a^ (5 µL)	[67]
Human whole blood (100 µL)	Protein precipitation with ACN	ESI-HPLC-QqQ-MS/MS (MRM)	82.0−103.0 nimodipine-*d_7_*	500 ^a^ (100 µL)	[68]
Human whole blood (100 µL)	Protein precipitation with ACN	ESI-HPLC-QqQ-MS/MS (SRM)	84.0−93.0 3-acetamidophenol	60 (10 µL)	[69]
Human whole blood (100 µL)	LLE (pH 9.2; butyl chloride: isopropanol)	ESI-HPLC-QqQ-MS/MS (MRM)	−92.0 to −96.0 MDMA-*d_5_*	100 (20 µL)	[70]
Human whole blood (1000 µL)	SPE	UHPLC-QqQ-MS/MS (MRM)	90.6−97.1 diazepam-*d_5_*	5 (1 µL)	[71]
Human whole blood (200 µL)	LLE (pH 9.0; ethyl acetate)	ESI-UHPLC-QqQ-MS/MS (MRM)	92.6−102.2 diclofenac-*d_4_*	0.5 (2 µL)	Presented method

─ Information not provided; Parameters expressed as: ^a^ lowest calibration level (LCL); ^b^ in units ng/g.

## Abbreviations

LOQ—Limit of quantification; HPLC—high performance liquid chromatography; UHPLC—ultra-high performance liquid chromatography; QqQ—triple quadrupole; IT—ion trap; MS/MS—tandem mass spectrometry; QTRAP—triple quadrupole linear ion trap; ESI—electrospray ionization; SRM—single reaction monitoring; MRM—multiple reaction monitoring; LLE—liquid–liquid extraction; SPE—solid phase extraction, ACN—acetonitrile; MeOH—methanol.
